# Patterns of Preoperative Tumor Markers Can Predict Resectability and Prognosis of Peritoneal Metastases: A Clustering Analysis

**DOI:** 10.1245/s10434-024-16860-y

**Published:** 2025-01-22

**Authors:** Malin Enblad, Peter Cashin, Lana Ghanipour, Wilhelm Graf

**Affiliations:** Department of Surgical Sciences, Colorectal Surgery, Uppsala University, Uppsala, Sweden

**Keywords:** Peritoneal metastases, Tumor markers, Clustering analysis, Cytoreductive surgery, HIPEC

## Abstract

**Background:**

Prediction of open–close and long-term outcome is challenging in patients undergoing cytoreductive surgery (CRS) and hyperthermic intraperitoneal chemotherapy (HIPEC). Prognostic scores often include factors not known at baseline. Therefore, we aimed to analyze whether patterns of preoperative tumor markers could aid in prediction of open–close surgery and outcome in patients with pseudomyxoma peritonei (PMP) or colorectal peritoneal metastases (PM).

**Patients and Methods:**

All patients accepted for CRS and HIPEC for PMP or colorectal PM at Uppsala University Hospital in 2013–2021 were included. The tumor markers CEA, CA19-9, CA125, CA72-4, and CA15-3 were clustered using the *k*-means algorithm; the average silhouette width determined the optimal numbers of clusters.

**Results:**

Clustering of patients with PMP (*n* = 138) and colorectal PM (*n* = 213) resulted in two clusters each. PMPCluster-1 (*n* = 124) had a 5-year overall survival (OS) of 77% (95% CI 69−85%), 11 (9%) open–close surgeries, and a median peritoneal cancer index (PCI) of 17. PMPCluster-2 (*n* = 14) patients had poorer prognosis (36%, 95% CI 15–85%, *p* = 0.003), more often open–close (*n* = 6, 43%, *p* = 0.002), and higher PCI (median 36, *p* < 0.001). ColorectalCluster-1 (*n* = 191) had a 5-year OS of 28% (95% CI 21–37%), median PCI of 11, and 38 (20%) open–close surgeries. ColorectalCluster-2 (*n* = 22) had poorer prognosis (10%, 95% CI 3–36%, *p* = 0.02), higher PCI (median 26, *p* < 0.001), higher completeness of cytoreduction score (*p* = 0.005), but no difference in open–close surgery (*n* = 6, 27%, *p* = 0.411). PMPCluster-2 and ColorectalCluster-2 were characterized by markedly elevated tumor markers. Open–close surgery was unusual in cases of normal CA72-4.

**Conclusions:**

Elevation of several preoperative tumor markers is associated with poor prognosis and increased risk of open–close. CA72-4 deserves increased attention.

**Supplementary Information:**

The online version contains supplementary material available at 10.1245/s10434-024-16860-y.

Cytoreductive surgery (CRS) and hyperthermic intraperitoneal chemotherapy (HIPEC) offer a potential cure or prolonged survival for patients with pseudomyxoma peritonei (PMP) or colorectal peritoneal metastases (PM). To achieve this, complete cytoreduction is needed.^1–3^ One of the main challenges is to estimate tumor burden and resectability before surgery. Open–close surgery, in which a patient undergoes only explorative laparotomy because an inoperable PM is found, occurs in about 20–30% of cases.^4–6^ This occurs despite extensive preoperative work-ups with computed tomography, positron emission tomography, and magnetic resonance imaging, as small PM are often overlooked.^7,8^ Explorative laparoscopy has been added to quantify the tumor burden before CRS and HIPEC, but assessment of patients with extensive PMP, or with previous surgery and adhesions, remains challenging.^5^

Prognosis varies among patients found eligible for CRS and HIPEC. The most established prognostic factors are peritoneal tumor burden, quantified intraoperatively at the beginning of CRS using the peritoneal cancer index (PCI),^9^ and completeness of cytoreduction score (CCS).^9^ However, these factors are not known before surgery, unlike the levels of the preoperative serum tumor markers carcinoembryonic antigen (CEA), cancer antigen (CA) 19-9, CA 125, CA72-4, and CA15-3. These are frequently elevated in PM and associated with prognosis,^10–13^ although these results have not significantly impacted the management of patients with PM.

Rather than analyzing individual tumor markers, this study aims to analyze patterns of preoperative tumor markers through clustering analysis. With this new approach, patients cluster into groups on the basis of similarities in tumor marker patterns. The purpose was to see whether these patterns could predict open–close surgery and survival in patients with PMP or colorectal PM.

## Patients and Methods

### Study Population

All patients assessed as suitable candidates and accepted for initial CRS and HIPEC for confirmed PMP or colorectal PM at Uppsala University Hospital, a Swedish center for surgical treatment of peritoneal surface malignancies, between 2013 and 2021, were included. Patients with other primary tumors (mesothelioma, small bowel adenocarcinoma, or ovarian cancer), repeated CRS, or no macroscopic PM at exploration (suspected PM, scheduled as potential HIPEC), were not included. Patients lacking preoperative tumor markers were excluded. The study was approved by the regional ethics committee (reference number 2013/203).

### Tumor Classification

The primary tumors and the PM were classified on the basis of primary tumor localization and in accordance with the World Health Organization (WHO) classification of tumors of the digestive system.^14^ Patients were divided into PMP and colorectal PM groups. The PMP group included all patients with appendiceal tumors, except those with non-mucinous adenocarcinoma, who were categorized into the colorectal PM group due to similarities in biological behaviors. The colorectal PM group included all patients with colorectal primary tumors. Patients with unknown exact localization of the primary tumor, but with the immunohistochemistry profile of colorectal cancer, were considered colorectal unless otherwise specified.

The histopathology of PMP was classified as mucinous carcinoma peritonei (MCP) grade 1–3, in accordance with the Peritoneal Surface Oncology Group International (PSOGI) classification. Patients with acellular mucin were reported separately.^15^ Colorectal PM was classified as adenocarcinoma (AC), mucinous adenocarcinoma (MAC), or signet ring cell carcinoma (SCC). Some patients with colorectal primary tumors had peritoneal disease classified in accordance with PSOGI by a pathologist, for example, MCP G2. These patients were classified as AC, MAC, or SCC, as the PSOGI classification is mainly for appendiceal tumors.

### CRS and HIPEC

Before being accepted for CRS and HIPEC, each patient was discussed at a multidisciplinary team conference. At the beginning of CRS, the peritoneal tumor burden is assessed and quantified using the PCI.^9^ The PCI is calculated by summing up lesion size scores (range 0–3) in 13 different abdominal regions, resulting in a total score of 1–39. The goal is to achieve complete cytoreduction to proceed with HIPEC, which means a CCS (range 0–3)^9^ of 0 in colorectal PM, although CCS 0–1 is considered acceptable in PMP. In this study, an uncertain CCS scoring, CCS 0–1, was considered CCS-1. CCS-2 often corresponds to debulking surgery and CCS-3 to open–close surgery. CCS-2 and CCS-3 were grouped together as open-close, and these patients did not receive HIPEC. The reason for open-close in CRS is most often extensive PM corresponding to a high PCI or PM involving liver hilum, large vessels, or extensive small bowel involvement.

HIPEC has been performed in accordance with the closed technique at our center since 2019,^16^ but the open Coliseum technique was used at the beginning of the study period.^16,17^ The choice of intraperitoneal chemotherapy depended mainly on the primary tumor or previous treatments. Mitomycin C was commonly used in PMP, and oxaliplatin and/or irinotecan was commonly used in colorectal PM.

### Tumor Markers

Tumor markers in blood were routinely analyzed at baseline, before CRS and HIPEC, and at the time of admission at Uppsala University Hospital. Tumor markers were not used to exclude patients from surgery.

#### CEA

CEA is a glycoprotein that derives its name from initially being detected only in cancer and embryonic tissue.^18^ It is localized to the apical surface of enterocytes and is the most commonly used tumor marker for colorectal cancer. Elevated levels are associated with a high tumor stage and tumor grade. Higher levels are seen in patients with left-sided tumors.^6^ High CEA is associated with poor prognosis after CRS and HIPEC in colorectal PM.^10,11^ The reference value was < 3.8 µg/L.

#### CA19-9

Cancer antigen 19-9 (CA19-9) is a glycoprotein mucin, elevated in gastrointestinal malignancies, and is most often analyzed in pancreatic cancer.^19^ CEA has been shown to be superior for response prediction in colorectal cancer,^12,20^ but high levels of CA19-9 have been strongly associated with a poor prognosis.^12^ It has also been independently associated with poor survival in PMP.^13^ The reference value was < 34 kU/L.

#### CA125

Cancer antigen 125 (CA125) is a glycoprotein that is elevated mainly in ovarian cancer, but all adenocarcinomas, especially when metastasized, can cause elevated levels.^21^ CA125 is sometimes elevated in PMP and levels can predict prognosis and resectability in both PMP^11,13^ and colorectal PM.^10^ The reference value was < 35 kU/L.

#### CA72-4

Cancer antigen 72-4 (CA72-4) is a glycoprotein analyzed in many different cancer forms, mainly gastric and ovarian cancer.^22^ In combination with CEA, it has the highest sensitivity for colorectal cancer,^23^ but is also often elevated in PMP.^11^ The reference value was < 6.9 kU/L.

#### CA15-3

Cancer antigen 15-3 (CA15-3) is elevated mainly in patients with breast cancer, but also in other malignancies.^24^ It is a mucinous glycoprotein, a product of the *mucin-1* gene, found in almost all epithelial cells.^25^ It has no crucial role in colorectal cancer or PMP prognostication, but has been found to return to normal levels after CRS in patients with PMP.^13^ The reference value was < 25 kU/L.

### Clustering Analysis

To perform an unsupervised classification of the patients on the basis of similarities and patterns of preoperative tumor markers, the *k*-means clustering algorithm was used and analyzed within R.^26,27^ Initially, other clustering methods were also tested, such as partitioning around medoids, hierarchical clustering, and fuzzy clustering, which all resulted in the same number of optimal clusters. *k*-Means clustering was chosen, as it is one of the most common and intuitive methods, grouping the patients with the shortest distance to the cluster center (centroid point) in a multidimensional space into a predefined number of groups (*k*). To ensure that the tumor markers contributed equally, despite different values, the tumor data were scaled before clustering. This was done by subtracting the mean and dividing by the standard deviation, so that each variable has a mean of 0 and a standard deviation of 1. The optimal number of *k* was determined using the average silhouette width.^28^ The starting centroid point was placed randomly and this was repeated 10,000 times. Principal component analysis was performed to reduce the dimensions of the data and visualize the clusters. This created linear combinations of the tumor markers with the first principal component derived in a way that maximized the explained variance. Each additional principal component was derived in the same manner and was orthogonal to the other principal components. The clusters were visualized in a scatterplot using the first two principal components as the coordinate system.

### Statistical Analysis

The distributions of tumor marker levels before clustering were illustrated with histograms using a logarithmic base 2 scale, with reference, median, and mean values as vertical lines. After clustering, the tumor marker levels were illustrated using heatmaps, where the color intensity increased with every percentile from the reference value. In tables, continuous data are presented as medians with interquartile ranges. Fisher’s exact test was used for comparisons of categorical data and the Mann–Whitney *U* test was used to compare continuous data. Overall survival (OS) was calculated from date of CRS to death from any cause using Kaplan–Meier and presented as cumulative proportion surviving. Patients alive were censored at last follow-up. Risk factors for poor survival were analyzed with univariate and multivariate Cox proportional hazard regression analyses and presented as hazard ratios (HRs) with 95% confidence intervals (CIs). Factors known before surgery were chosen and CCS was therefore not included. However, true PCI and histopathology were also included, as preoperative radiology often indicates if the tumor burden is extensive, and histopathology is sometimes known preoperatively and associated with prognosis. The tumor markers were analyzed individually in one analysis and as clusters in one analysis. All variables in the univariate analyses were included in the multivariate analysis. The risk of open–close surgery was analyzed using univariate logistic regression and presented with odds ratios (ORs) with 95% CIs. A *p*-value of < 0.05 was considered statistically significant. R version 4.2.2 (R Foundation for Statistical Computing, Vienna, Austria) was used for statistical analyses.

## Results

### Study Population

After exclusion of patients with other primary tumors (*n* = 40), repeated CRS (*n* = 21), patients with no macroscopic PM at exploration (*n* = 74), patients lacking preoperative tumor markers (*n* = 14), and non-Swedish citizens without follow-up data (*n* = 2), 351 patients were included. The patients were grouped as PMP (*n* = 138) or colorectal PM (*n* = 213) and analyzed separately. Baseline clinical characteristics of the patients are presented in Table 1. The distributions of tumor marker levels for patients with PMP and colorectal PM are shown in Supplementary Figs. 1 and 2, respectively. The largest difference between patients with PMP and colorectal PM was that patients with PMP had a higher median CA72-4 (27 versus 7 kU/L).

**Table 1:** Characteristics of patients with pseudomyxoma peritonei or colorectal peritoneal metastases, with intention to treat with cytoreductive surgery and hyperthermic intraperitoneal chemotherapy, 2013–2021

Characteristics	PMP *n* = 138 *n* (%)	Colorectal PM *n* = 213 *n* (%)
Sex		
Male	72 (52)	95 (45)
Female	66 (48)	118 (55)
Age, years (median, IQR)	62 (50–71)	66 (54–71)
Primary tumor localization		
Appendix	137 (99)	5 (2)
Right colon	0 (0)	102 (48)
Left colon	0 (0)	67 (31)
Rectum	0 (0)	26 (12)
Colorectal NOS	1 (1)	13 (6)
Neoadjuvant treatment	4 (3)	45 (22)
PCI (median, IQR)	20 (9–31)	12 (6–24)
CCS		
0	81 (59)	160 (75)
1	40 (28)	9 (4)
2/3 (open–close)	17 (12)	44 (21)
Histopathology PM		
Acellular mucin	25 (18)	0 (0)
PSOGI MCP G1	39 (29)	0 (0)
PSOGI MCP G2	47 (34)	0 (0)
PSOGI MCP G3	22 (16)	0 (0)
Adenocarcinoma	0 (0)	115 (54)
Mucinous adenocarcinoma	0 (0)	60 (28)
Signet ring cell carcinoma	0 (0)	26 (12)
No neoplastic cells	5 (4)	11 (5)

*PMP* pseudomyxoma peritonei, *PM* peritoneal metastases, *IQR* interquartile range, *NOS* not otherwise specified, *PCI* peritoneal cancer index, *CCS* completeness of cytoreduction score, *PSOGI MCP G* Peritoneal Surface Oncology Group International classification of mucinous carcinoma peritonei grade 1–3

### Clustering PMP

**Fig 1:**
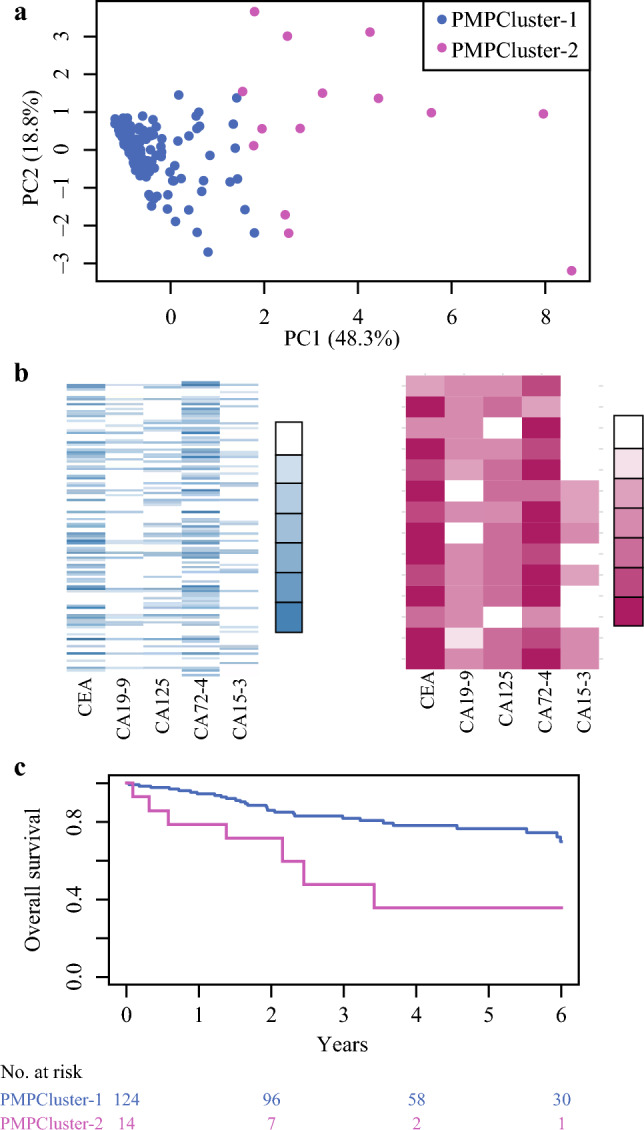
**a** Scatterplot of patients with pseudomyxoma peritonei separated into two groups on the basis of clustering of tumor markers; **b** heatmaps of tumor marker levels for the two clusters, white indicates levels below the reference value and color intensity increases with each percentile of elevation from the reference value; **c** overall survival Kaplan–Meier curves and number at risk table for the two clusters; *PC* principal component, *PMP* pseudomyxoma peritonei

The silhouette method was used to determine the optimal number of clusters with the least overlap, which was two. Clustering on the basis of tumor marker levels resulted in PMPCluster-1 (*n* = 124) and PMPCluster-2 (*n* = 14, Fig. 1a). The distributions of tumor marker levels in each group are shown in Fig. 1b. PMPCluster-2 patients differed from PMPCluster-1 patients by having generalized elevation of tumor markers. The heatmaps illustrate that all patients in PMPCluster-2 had at least three elevated tumor markers, the majority with four or five markers elevated to very high levels. All patients in PMPCluster-2 had elevated CEA and CA72-4 (Fig. 1b).

PMPCluster-1 patients had a better prognosis, with a 5-year OS of 77% (95% CI 69–85%), compared with 36% (95% CI 15–85%, *p* = 0.003) in PMPCluster-2 (Fig. 1c). When open–close patients were excluded, PMPCluster-1 patients had a 5-years OS of 82% (95% CI 74–100%) and PMPCluster-2 patients 31% (95% CI 7–100%, *p* = 0.002). The two clusters also had significant differences in clinical and tumor-related characteristics (Table 2). Patients in PMPCluster-2 were younger, had higher PCI, and more aggressive histopathology, with few patients having acellular mucin or PSOGI MCP G1. The patients with acellular mucin (*n* = 25) or with no neoplastic cells found in peritoneal resection specimens (*n* = 5) were all in PMPCluster-1. In PMPCluster-2, CA15-3 was the only tumor marker that was not elevated compared with the reference value.

**Table 2:** Characteristics of patients with pseudomyxoma peritonei after clustering into two groups on the basis of preoperative tumor marker levels

Characteristics	PMPCluster-1 *n* = 124 *n* (%)	PMPCluster-2 *n* = 14 *n* (%)	*p-*Value
Sex			
Male	66 (53)	6 (43)	0.576
Female	58 (47)	8 (57)	
Age, years	63 (53–71)	57 (43–64)	0.061
PCI (median, IQR)	17 (8–27)	36 (33–39)	< 0.001
Open–close	11 (9)	6 (43)	0.002
CCS			< 0.001
0	79 (64)	2 (14)	
1	34 (27)	6 (43)	
2/3 (open–close)	11 (9)	6 (43)	
Histopathology PM			0.057
Acellular mucin	25 (20)	0 (0)	
PSOGI MCP G1	38 (31)	2 (14)	
PSOGI MCP G2	39 (31)	9 (64)	
PSOGI MCP G3	17 (14)	3 (21)	
No neoplastic cells	5 (4)	0 (0)	
Tumor markers (median, IQR)			
CEA	4 (2–10)	118 (34–222)	< 0.001
CA19-9	12 (6–32)	885 (354–1919)	< 0.001
CA125	24 (14–47)	207 (113–403)	< 0.001
CA72-4	20 (3–61)	224 (108–556)	< 0.001
CA15-3	19 (13–25)	25 (22–32)	0.017

*IQR* interquartile range, *PCI* peritoneal cancer index, *CCS* completeness of cytoreduction score, *PM* peritoneal metastases, *PSOGI MCP G* Peritoneal Surface Oncology Group International classification of mucinous carcinoma peritonei grade 1–3

### Clustering Colorectal PM

**Fig. 2:**
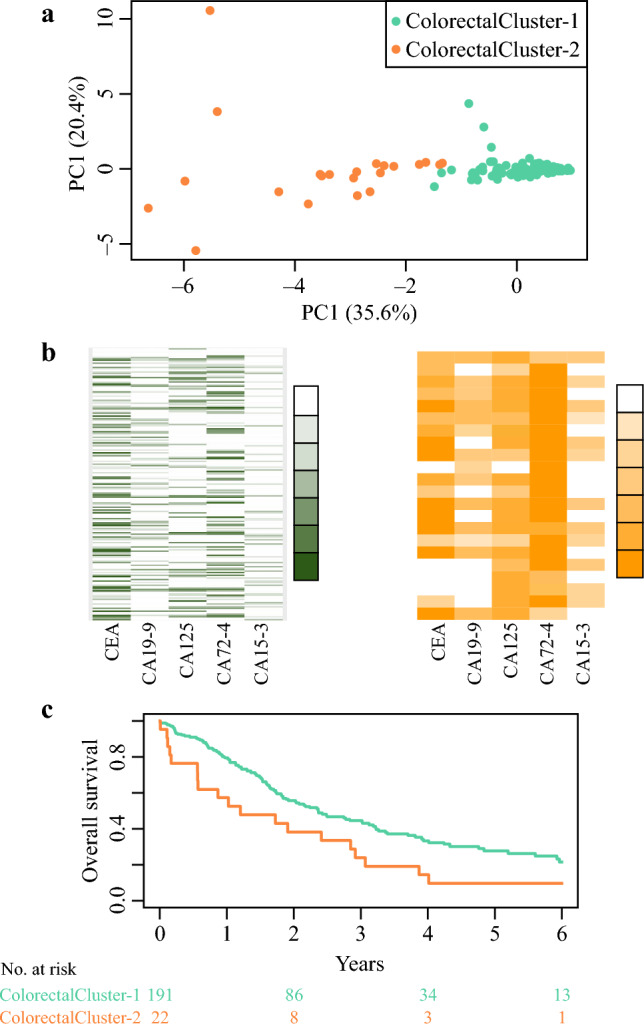
**a** Scatterplot of patients with colorectal peritoneal metastases separated into two groups on the basis of clustering of tumor markers; **b** heatmaps of tumor marker levels for the two clusters, white indicates levels below reference value and color intensity increases with each percentile of elevation from the reference value; **c** overall survival Kaplan–Meier curves and number at risk table for the two clusters; *PC* principal component

Two clusters were also optimal for colorectal PM, and clustering resulted in ColorectalCluster-1 (*n* = 191) and ColorectalCluster-2 (*n* = 22, Fig. 2a). ColorectalCluster-2 had markedly elevated levels of several tumor markers. CA72-4 was elevated in all patients, and CA125 in all but one patient (Fig. 2b).

The prognosis was poor in ColorectalCluster-2, with a 5-year OS of 10% (95% CI 3–36%), compared with 28% (95% CI 21–37%, *p* = 0.02) in ColorectalCluster-1 (Fig. 2c). When open–close patients were excluded, ColorectalCluster-2 patients had a 5-year OS of 13% (95% CI 4–48%) and ColorectalCluster-1 patients 34% (95% CI 26–44%, *p* = 0.1).

Clinical differences between the clusters indicated higher PCI and higher CCS in ColorectalCluster-2. There was no significant difference in open–close frequency. ColorectalCluster-2 tended to have more mucinous/signet ring adenocarcinomas (Table 3). Two patients in ColorectalCluster-2 had no neoplastic cells in peritoneal resections, both of whom had locally advanced colon tumors with mucinous histopathology and radiology with suspected local carcinomatosis. Perioperatively, uncertain macroscopic findings near the tumor (corresponding to PCI 2 and 3, respectively) were not confirmed as malignancies during the histopathological examination.

**Table 3:** Characteristics of patients with colorectal peritoneal metastases after clustering into two groups on the basis of preoperative tumor marker levels

Characteristics	ColorectalCluster-1 *n* = 191 *n* (%)	ColorectalCluster-2 *n* = 22 *n* (%)	*p*-Value
Sex			0.259
Male	88 (46)	7 (31)	
Female	103 (54)	15 (68)	
Age, years (median, IQR)	66 (55–71)	61 (50–68)	0.062
PCI (median, IQR)	11 (6–22)	26 (22–32)	< 0.001
Open–close	38 (20)	6 (27)	0.411
CCS			0.005
0	148 (77)	12 (55)	
1	5 (3)	4 (18)	
2/3 (open–close)	38 (20)	6 (27)	
Histopathology PM			*0.073*
Acellular mucin	0 (0)	0 (0)	
Adenocarcinoma	108 (57)	7 (32)	
Mucinous adenocarcinoma	52 (27)	8 (36)	
Signet ring cell carcinoma	21 (11)	5 (23)	
No neoplastic cells	9 (5)	2 (9)	
Tumor markers (median, IQR)			
CEA	5 (2–19)	36 (11–91)	< 0.001
CA19-9	16 (7–54)	102 (24–440)	< 0.001
CA125	20 (10–47)	226 (108–408)	< 0.001
CA72-4	6 (2–19)	600 (283–600)	< 0.001
CA15-3	18 (12–24)	29 (29–36)	< 0.001

*IQR* interquartile range, *PCI* peritoneal cancer index, *CCS* completeness of cytoreduction score, *PM* peritoneal metastases, *PSOGI MCP G* Peritoneal Surface Oncology Group International classification of mucinous carcinoma peritonei grade 1–3

### Risk Factors—PMP

Univariate Cox proportional hazard regression analysis for OS was performed and all tumor markers, except CA15-3, were found to be associated with poor survival, as were PCI and PSOGI MCP G3 histopathology. In the multivariate analysis of the individual tumor markers, only CA72-4 was independently associated with poor survival. When analyzing tumor markers by clusters; PMPCluster-2 was independently associated with poor prognosis (HR 4.29, 95% CI 4.29–13.16), together with age, PCI, and PSOGI MCP G3 (Supplementary Table 1). PMPCluster-2 was also a univariate risk factor for open–close surgery (OR 7.70, 95% CI 2.26–26.3, Supplementary Table 2).

### Risk Factors—Colorectal PM

Among patients with colorectal PM, ColorectalCluster-2 was associated with poor survival in the univariate analysis, as were all tumor markers except CA15-3. Mucinous histopathology was associated with better survival, and signet ring cell carcinoma was associated with poor survival. In the multivariate analysis, ColorectalCluster-2 was not significantly associated with poor survival, whereas CEA and CA19-9 were (Supplementary Table 3). ColorectalCluster-2 was not associated with open–close surgery (OR 1.51, 95% CI 0.55–4.12, Supplementary Table 2).

### CA72-4

**Fig. 3:**
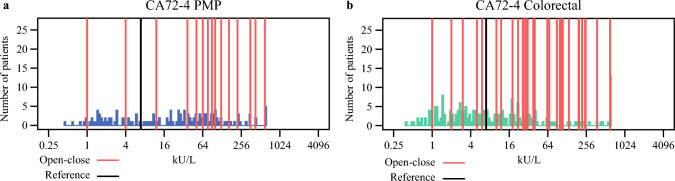
Distribution of preoperative CA72-4 values; values for patients with open–close laparotomy is indicated by red lines and reference value is indicated by black lines; **a** pseudomyxoma peritonei and **b** colorectal peritoneal metastases

Since CA72-4 was elevated in all patients with PMPCluster-2 and ColorectalCluster-2, the role of normal CA72-4 was further examined. Open–close was uncommon in cases of normal CA72-4 and occurred in 2 (4%) of patients with PMP (Fig. 3a) and 5 (5%) patients with colorectal PM (Fig. 3b).

## Discussion

Prediction of open–close surgery and identification of patients who will benefit from CRS and HIPEC remain major challenges. In this study, a clustering analysis of preoperative tumor markers demonstrated that patients with PMP and colorectal PM who had elevated levels of several preoperative tumor markers had poorer prognoses. For PMP, this was also associated with an increased risk of open–close surgery. In addition, CA72-4, a tumor marker that has not received much previous attention, was markedly elevated in all patients in the clusters with the poorest prognosis.

Despite advances in the preoperative assessment of patients with PMP or colorectal PM, open–close surgery occurs in about 20–30%.^4–6^ This is a devastating experience for patients and also results in loss of healthcare resources. In this study of consecutive patients judged eligible for CRS and HIPEC, 17% underwent open–close surgery. Furthermore, the prognosis of patients undergoing full treatment with CRS and HIPEC is hard to determine, and molecular factors may play a large role in future prognostication. In the meantime, validated prognostic scores for colorectal PM, such as the peritoneal surface disease severity score (PSDSS)^29^ and the colorectal peritoneal metastases prognostic surgical score (COMPASS),^30^ are used. Prediction is based on age, logo-regional lymph nodes, PCI, and presence of signet ring cells. The colorectal peritoneal score (COREP)^31^ and the modified COREP (mCOREP) also include tumor markers. However, although the same variables are considered to be risk factors in PMP, the biological behaviors differ, and PCI can be very high in all PSOGI MCP grades.

Unlike the above-mentioned risk factors, tumor markers are available before CRS and present in many different malignancies. Due to the heterogeneity of PM, their predictive and prognostic role in PM is not easy to study or interpret, and use differs between HIPEC center. Because the tumor markers in the present study have all been associated with poor prognosis^10–13^—or, in the case of CA15-3, return to normal levels after CRS and HIPEC^13^—they have been routinely sampled preoperatively and during follow-up to diagnose early recurrences. Despite the prognostic association, none of the tumor markers are currently taken into account for treatment strategy or patient information before CRS and HIPEC.

In this study, clustering analysis was used to explore patterns of preoperative tumor markers without adding additional factors beyond dividing the patients into PMP and colorectal PM groups. *k*-Means clustering was chosen as it is one of the most common clustering methods, and the optimal number of clusters is calculated to create minimal overlap.^26–28^ In both PMP and colorectal PM, two groups were statistically optimal. The small number of clusters is statistically explained by large variations in tumor marker levels and patterns of elevations. If creating additional groups, the clusters overlap too much in regard to tumor marker levels and patterns. Thus, despite the fact that this study did not reveal several distinct groups with different tumor marker patterns, the clustering confirmed the large heterogeneity in tumor marker elevations and highlights how difficult it is to interpret tumor markers.

In PMP, clustering identified PMPCluster-2 with poor prognosis, high PCI, aggressive histopathology, and with at least three elevated tumor markers. Similar results have been demonstrated previously, i.e., the more tumor markers that are elevated, the worse the prognosis for the patient.^11,32^ In addition, inclusion in PMPCluster-2 was an independent risk factor for open–close surgery. When the open–close patients were excluded, PMPCluster-2 still had a poorer prognosis. The high risk of open–close surgery and poor prognosis associated with marked elevations of several tumor markers can be incorporated into the preoperative assessment, and also included in the information given to the patient preoperatively.

In colorectal PM, ColorectalCluster-2 was characterized by extremely high levels of several tumor markers and a strikingly poor prognosis, with a 5-year OS of 10%. It was also associated with high CCS. CCS-1 is considered an insufficient oncological result and a strong negative prognostic factor in colorectal PM.^33^ In addition, ColorectalCluster-2 patients had higher PCI and more aggressive histopathology. However, despite the very poor prognosis in ColorectalCluster-2, open–close surgery was not significantly more common in this group, and the survival of patients undergoing full treatment with CRS and HIPEC was not significantly poorer. This could be explained by a higher degree of inoperable disease without high tumor marker levels. In addition, patients with colorectal PM have poorer prognosis in general, and any potential differences would require higher statistical power for detection.

This study also demonstrated that CA72-4 is a tumor marker that is elevated in both PMP and colorectal PM; is associated with open–close surgery and poor prognosis; and is independently associated with poor prognosis in PMP. Despite CA72-4 having high sensitivity for colorectal cancer,^23^ it is mainly analyzed in gastric and ovarian cancer. In gastric cancer, it has the second highest sensitivity for PM and the highest sensitivity for ovarian metastases.^34^ A combination of elevated CEA and CA72-4 in peritoneal fluids is associated with PM. Studies of CA72-4 in PMP and colorectal PM are lacking, but CA72-4 clearly exhibits properties that deserve increased attention. The finding that open–close laparotomy is unusual in patients with normal CA72-4 is clinically useful, and in borderline situations, a normal CA 72-4 could speak in favor of a surgical exploration. Conversely, CA15-3 was found to have a limited role in PMP and colorectal PM, and can be excluded from preoperative analyses.

This study has some noteworthy limitations. First, clustering is a method that analyzes values from a specific study population. Changes in group compositions, for example by excluding a patient, may affect the distance between tumor marker levels and the clustering result. This makes it difficult to reproduce exact results. However, this clustering study revealed that marked elevation of several tumor markers was a shared characteristic of PMPCluster-2 and ColorectalCluster-2, which could be reproduced in prospective cohorts to validate the current findings. Second, tumor markers were scaled to be comparable. This may have affected the impact of the individual tumor markers. In addition, 14 patients were lacking information on tumor markers and they were excluded from the study. Third, the study population was too small for detection of any differences in the colorectal PM group, and PMP Cluster-1 included only 14 patients. The small sample sizes may affect generalizability and interpretation of the differences between groups. Lastly, this is a study based on historical data with the risk of temporal changes in overall management and interpretation. However, the most important information regarding tumor markers, open–close surgery, and survival, is of high quality.

In conclusion, markedly elevated levels of several preoperative tumor markers are associated with increased risk of open–close surgery, high-risk features, and poor prognosis, especially in patients with PMP. This should be taken into account in the overall assessment and the preoperative information given to patients before CRS and HIPEC. In addition, elevated CA72-4 deserves increased attention as a potential marker of poor prognosis, and oppositely, normal values indicate a low risk of open–close laparotomy.

## Supplementary Information

Below is the link to the electronic supplementary material.

Supplementary file1 (JPG 141 KB)

Supplementary file2 (JPG 139 KB)

Supplementary file3 (DOCX 16 KB)

Supplementary file4 (DOCX 16 KB)

Supplementary file5 (DOCX 16 KB)
