# Supplementary figures and images for: Patterns of Preoperative Tumor Markers Can Predict Resectability and Prognosis of Peritoneal Metastases: A Clustering Analysis
Source: Ann Surg Oncol. 2025 Jan 22;32(5):3638–47. doi: 10.1245/s10434-024-16860-y (PMC11976843; doi:10.1245/s10434-024-16860-y)

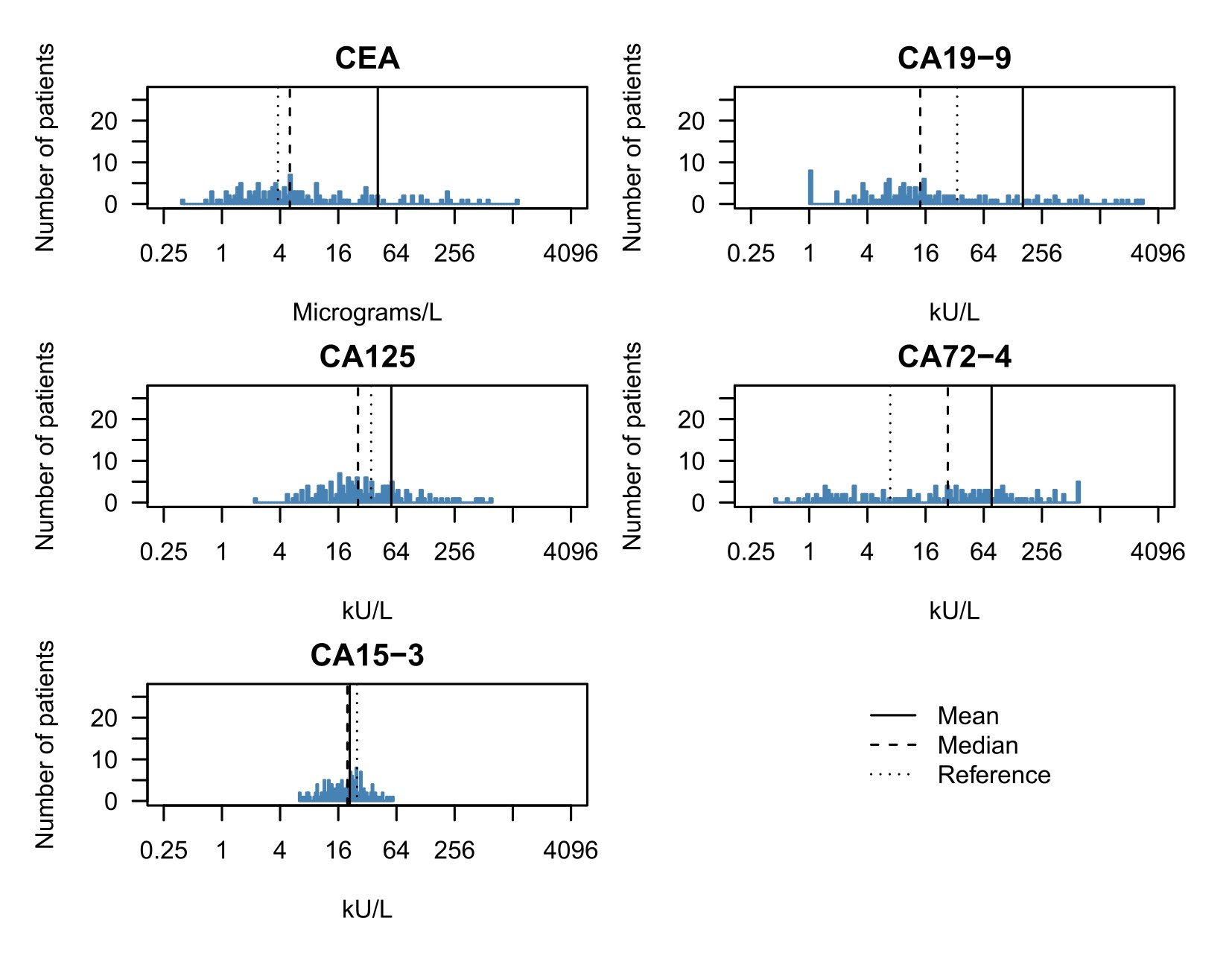

Supplement: Supplementary file 1 — Supplementary file1 (JPG 141 KB) [file 10434_2024_16860_MOESM1_ESM.jpg]

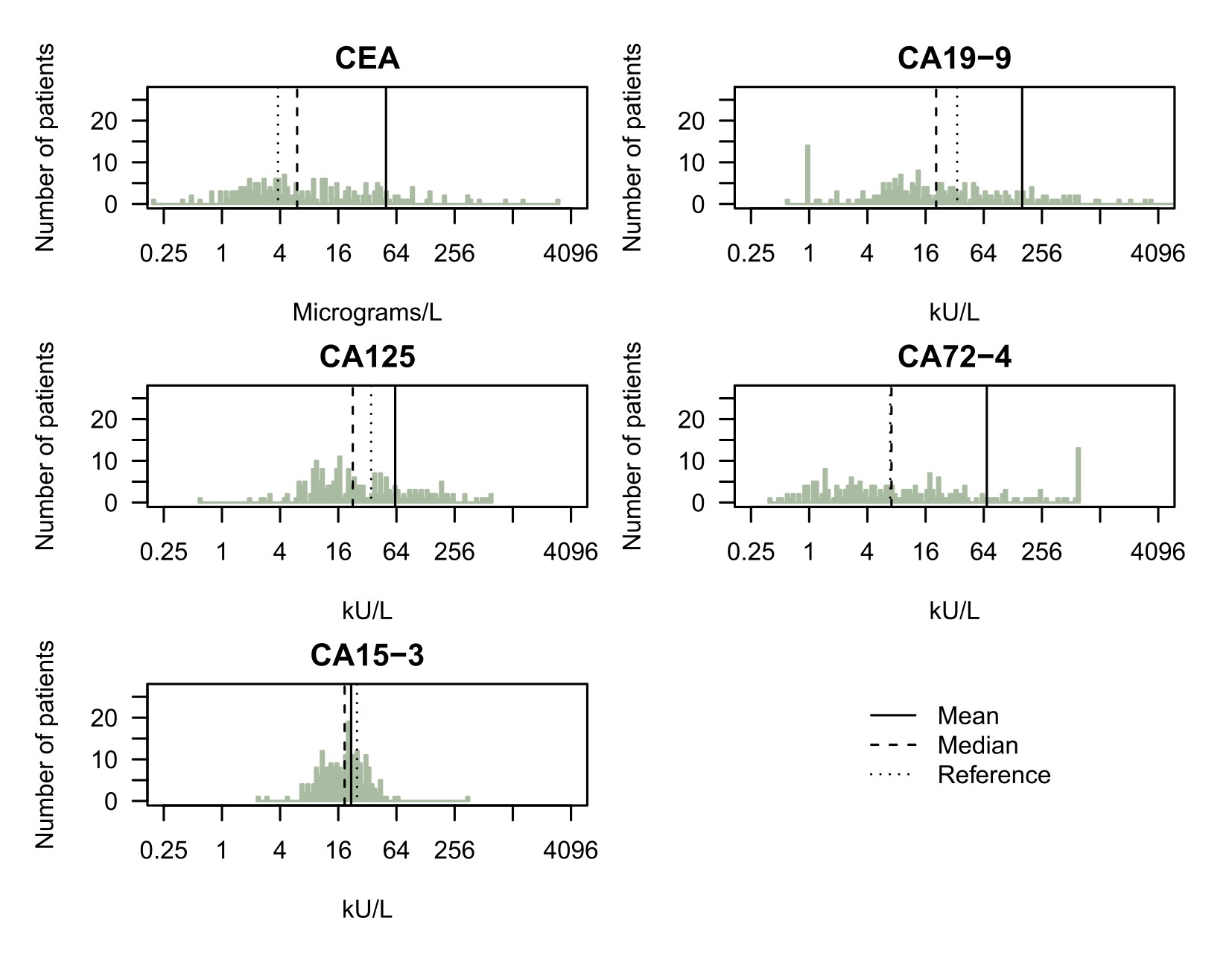

Supplement: Supplementary file 2 — Supplementary file2 (JPG 139 KB) [file 10434_2024_16860_MOESM2_ESM.jpg]
