# Supplementary material for: Patterns of Preoperative Tumor Markers Can Predict Resectability and Prognosis of Peritoneal Metastases: A Clustering Analysis
Source: Ann Surg Oncol. 2025 Jan 22;32(5):3638–47. doi: 10.1245/s10434-024-16860-y (PMC11976843; doi:10.1245/s10434-024-16860-y)
Supplement: Supplementary file 3 — Supplementary file3 (DOCX 16 KB) [file 10434_2024_16860_MOESM3_ESM.docx]

| **Supplementary table 1.** Univariate and multivariate Cox proportional regression hazard analysis for risk of death in patients with pseudomyxoma peritonei. Tumour markers were analysed individually and as clusters. | | | |
| --- | --- | --- | --- |
|  |  | INDIVIDUAL TUMOUR MARKERS | CLUSTERS |
|  | OS  Univariate  HR (95% CI) | OS  Multivariate  HR (95% CI) | OS  Multivariate  HR (95% CI) |
| Sex | 0.65 (0.34–1.26) | 0.77 (0.33–1.80) | 0.94 (0.44–2.04) |
| Age | 1.02 (0.99–1.05) | 1.03 (0.99–1.06) | **1.04 (1.00–1.07)** |
| PCI | **1.05 (1.02–1.08)** | 1.02 (0.97–1.08) | **1.05 (1.00–1.11)** |
| Histopathology PM |  |  |  |
| Acellular mucin | 0.40 (0.09–1.91) | 0.72 (0.14–1.91) | 0.67 (0.14–3.33) |
| PSOGI MCP G1 | 1.00 | 1.00 | 1.00 |
| PSOGI MCP G2 | 1.21 (0.47–3.08) | 0.76 (0.27–2.15) | 0.72 (0.26–1.99) |
| PSOGI MCP G3 | **8.98 (3.71–21.74)** | **12.03 (4.38–33.05)** | **12.01 (4.64–31.09)** |
| No neoplastic cells | 0.79 (0.01–6.34) | 2.63 (0.25–27.42) | 3.86 (0.37–40.78) |
| Tumour markers |  |  |  |
| CEA | **1.003 (1.001–1.005)** | 1.001 (0.999–1.003) |  |
| CA19-9 | **1.001 (1.000–1.001)** | 1.000 (0.999–1.001) |  |
| CA125 | **1.004 (1.002–1.006)** | 1.001 (0.997–1.004) |  |
| CA72-4 | **1.004 (1.003–1.006)** | **1.004 (1.001–1.007)** |  |
| CA15-3 | 0.99 (0.96–1.02) | 0.97 (0.938–1.010) |  |
| Clusters |  |  |  |
| PMPCluster-1 | 1.00 |  | 1.00 |
| PMPCluster-2 | **3.35 (1.45–7.74)** |  | **4.29 (1.40–13.16)** |
|  |  |  |  |
| OS; overall survival, HR; hazard ratio, CI; confidence interval, PCI; peritoneal cancer index, PM; peritoneal metastases, PSOGI MCP G; Peritoneal Surface Oncology Group International classification of mucinous carcinoma peritonei Grade 1–3, PMP; pseudomyxoma peritonei | | | |
