# Supplementary material for: Patterns of Preoperative Tumor Markers Can Predict Resectability and Prognosis of Peritoneal Metastases: A Clustering Analysis
Source: Ann Surg Oncol. 2025 Jan 22;32(5):3638–47. doi: 10.1245/s10434-024-16860-y (PMC11976843; doi:10.1245/s10434-024-16860-y)
Supplement: Supplementary file 4 — Supplementary file4 (DOCX 16 KB) [file 10434_2024_16860_MOESM4_ESM.docx]

| **Supplementary table 2.** Univariate logistic regression analyses of tumour markers for the risk of open-close surgery in patients with pseudomyxoma peritonei or colorectal peritoneal metastases. | | | | |
| --- | --- | --- | --- | --- |
|  | PMP |  | Colorectal PM |  |
|  | OR (95% CI) | *p-value* | OR (95% CI) | *p-value* |
| Tumour markers |  |  |  |  |
| CEA | **1.01 (1.00–1.01)** | ***0.001*** | 1.00 (0.99–1.00) | *0.133* |
| CA19-9 | **1.00 (1.00–1.00)** | ***0.021*** | **1.00 (1.00–1.00)** | ***0.029*** |
| CA125 | **1.00 (1.00–1.01)** | ***0.045*** | **1.00 (1.00–1.00)** | ***0.014*** |
| CA72-4 | **1.00 (1.00–1.01)** | ***0.043*** | **1.00 (1.001–1.007)** | ***0.034*** |
| CA15-3 | 1.01 (0.96–1.06) | *0.754* | 1.00 (0.99–1.01) | *0.744* |
| Clusters |  |  |  |  |
| Cluster-1 | 1.00 |  | 1.00 |  |
| Cluster-2 | **7.70 (2.26–26.30)** | ***0.001*** | 1.51 (0.55–4.12) | *0.421* |
|  |  |  |  |  |
| PMP; pseudomyxoma peritonei, PM; peritoneal metastases, OR; odds ratio, CI; confidence interval. | |  |  |  |
