# Supplementary material for: Patterns of Preoperative Tumor Markers Can Predict Resectability and Prognosis of Peritoneal Metastases: A Clustering Analysis
Source: Ann Surg Oncol. 2025 Jan 22;32(5):3638–47. doi: 10.1245/s10434-024-16860-y (PMC11976843; doi:10.1245/s10434-024-16860-y)
Supplement: Supplementary file 5 — Supplementary file5 (DOCX 16 KB) [file 10434_2024_16860_MOESM5_ESM.docx]

| **Supplementary table 3**. Univariate and multivariate Cox proportional regression hazard analysis for risk of death in patients with colorectal peritoneal metastases. Tumour markers were analysed individually and as clusters. | | | |
| --- | --- | --- | --- |
|  |  | INDIVIDUAL TUMOUR MARKERS | CLUSTERS |
|  | OS  Univariate  HR (95% CI) | OS  Multivariate  HR (95% CI) | OS  Multivariate  HR (95% CI) |
| Sex | 0.82 (0.59–1.15) | 1.07 (0.74–1.54) | 0.99 (0.69–1.42) |
| Age | 1.00 (0.99–1.02) | 1.00 (0.99–1.02) | 1.00 (0.99–1.02) |
| PCI | **1.07 (1.05–1.09)** | **1.07 (1.05–1.09)** | **1.07 (1.05–1.09)** |
| Histopathology PM |  |  |  |
| Adenocarcinoma | 1.00 | 1.00 | 1.00 |
| Mucinous adenocarcinoma | **0.62 (0.41–0.94)** | **0.43 (0.27–0.67)** | **0.47 (0.30–0.72)** |
| Signet ring cell carcinoma | **2.12 (1.32–3.41)** | 0.92 (0.50–1.66) | 0.79 (0.44–1.41) |
| No neoplastic cells | **0.19 (0.06–0.62)** | 0.37 (0.11–1.24) | 0.33 (0.10–1.10) |
| Tumour markers |  |  |  |
| CEA | **1.001 (1.000–1.001)** | **1.001 (1.000–1.002)** |  |
| CA19-9 | **1.001 (1.000–1.001)** | **1.000 (1.000–1.000)** |  |
| CA125 | **1.002 (1.001–1.004)** | 1.000 (0.999–1.003) |  |
| CA72-4 | **1.002 (1.001–1.002)** | 0.999 (0.999–1.000) |  |
| CA15-3 | 1.000 (0.990–1.004) | 0.997 (0.986–1.007) |  |
| Clusters |  |  |  |
| ColorectalCluster-1 | 1.00 |  | 1.00 |
| ColorectalCluster-2 | **1.81 (1.11–2.93)** |  | 1.00 (0.57–1.77) |
|  |  |  |  |
| OS; overall survival, HR; hazard ratio, CI; confidence interval, PCI; peritoneal cancer index, PM; peritoneal metastases | | | |
